# Goal adjustment processes as coping responses to a blocked goal: the sample case of ostracism

**DOI:** 10.3389/fpsyg.2025.1531759

**Published:** 2025-04-08

**Authors:** Farina Rühs, Werner Greve, Cathleen Kappes

**Affiliations:** ^1^Institute of Psychology, University of Hildesheim, Hildesheim, Germany; ^2^Criminological Research Institute of Lower Saxony, Hannover, Germany

**Keywords:** ostracism, self-regulation, goal adjustment, goal disengagement, Cyberball, coping

## Abstract

**Introduction:**

Recently, Rühs et al. (2022) used an adapted ostracism-paradigm to study goal adjustment processes, and goal disengagement processes (GD) in particular, as regulatory responses to goal-blocking situations such as ostracism. The present study conceptually replicates this study and extends it by inclusion of sub-personal indicators of GD in the paradigm.

**Methods:**

The goal to belong to a newly formed group was induced in 188 participants (Induction Phase). Afterwards, blockage of this goal was experimentally manipulated via ostracism: Participants were either included or excluded from their group in a virtual ball game (Cyberball, Blockage Phase). Finally, participants worked alone on a cognitive task to give regulatory responses some time to unfold. After each phase, dependent measures were recorded (e.g., indicators of GD and well-being).

**Results:**

Exclusion (vs. inclusion) in Cyberball lead to a decrease in subjective attainability of the belonging goal (goal blockage) and to affective-cognitive and behavioral GD (e.g., explicit devaluation of the belonging goal and the own group, behavioral deprioritization of ostracizing compared to new group members in a following game). However, ostracism had no effect on implicit group evaluation (repeated IATs showed a constant own group bias) and although excluded participants recovered from ostracism-induced impairments in emotions and needs, associations between recovery and GD indicators were mixed.

**Discussion:**

Most of the results of Rühs et al. (2022) could be replicated. Beyond that, the present study showed divergence of personal and sub-personal indicators of cognitive-affective GD (i.e., change in explicit and implicit group evaluations). This illustrates the importance of combining personal and sub-personal perspectives in GD research. Taken together, the study contributes to a conceptual and functional clarification of GD processes and, at the same time, offers a fruitful new perspective on coping with ostracism.

## 1 Introduction

Imagine you are standing in a group of people who have just met at a seminar and during the break, they talk about going out together in the evening, but do not invite you to join them. Or imagine that small groups are formed during the warm-up for sports exercises, but you are completely ignored when the groups are formed. Being socially excluded can be painful. Most people experience this several times in their lives (Rudert et al., 2020), very overtly through negative attention (e.g., communicated dislike and explicit exclusion) or more subtly through being ignored (*ostracism*, e.g., silent treatment). Experiences of social exclusion threaten the fundamental need to belong (Baumeister and Leary, 1995) and other fundamental human needs such as the need for self-esteem, the need for control and the need for meaningful existence (Williams, 2009). In short, exclusion is a threat to the self. Moreover, experiencing social exclusion is associated with other negative outcomes, ranging from intrapsychic symptoms (e.g., worsened affective experience and reduced self-esteem) to interpersonal problems (e.g., antisocial behavior or withdrawal; for meta-analyses/reviews see, e.g., Blackhart et al., 2009; Gerber and Wheeler, 2009; Leary, 2015; Williams, 2007) and even aggression and radicalization (e.g., Pfundmair et al., 2024; Ren et al., 2018). Thus, experiences of social exclusion require regulatory responses from the threatened individual to stabilize the self and maintain or regain well-being.

Research based on the *two-process model of developmental regulation* (Brandtstädter and Rothermund, 2002) has emphasized that *goal adjustment processes* (e.g., disengagement from blocked goals and reengagement in new, more attainable ones) contribute to such stabilizing in self-threatening situations associated with goal-blocking experiences. Ostracism can be considered a prototype of such situations. Rühs et al. (2022) found that ostracism actually causes the experience of being blocked from pursuing current goals (particularly the goal to belong to the group from which one was excluded). However, despite numerous studies on the consequences of ostracism and related coping-processes (for an overview, see, e.g., Williams and Nida, 2017) goal adjustment processes have only recently been studied in the context of ostracism. Rühs et al. (2022) used an adaptation of a typical ostracism paradigm (Cyberball: Williams and Jarvis, 2006) to investigate *goal disengagement* (GD) processes (as one important family of goal adjustment processes) in response to blockage of the goal to belong. In this study ostracism led to decreased subjective attainability of the goal to belong to the group (indicating goal blockage) and triggered cognitive-affective (e.g., reevaluation processes) and behavioral (e.g., changes in behavioral preferences) processes of disengagement from this goal.

The present study aimed, first, to conceptually replicate the findings of Rühs et al. (2022), but also, second, to extend them to other indicators of GD, and, third, to investigate the indicators’ associations and their potential adaptive function in more depth. The most substantial extension compared to the original study is the inclusion of *sub-personal* indicators of GD in the paradigm. We use the term “sub-personal” in contrast to “personal” to differentiate between two explanatory stances from which psychological processes can be approached (see also Greve and Wentura, 2007; Rothermund et al., 2020). Personal psychology (*intentional stance*) uses reasons to describe and explain people’s actions (e.g., a person no longer participates in group tasks because the goal to belong to the group has lost importance to the person). Sub-personal psychology (for instance, *design stance*), on the other hand, investigates the causal mechanisms underlying people’s behavior beyond intentionality (e.g., a person responds behaviorally slower to stimuli associated with a particular group membership because positive associations with that group membership in cognitive networks have become weaker). This distinction has many implications for basic scientific assumptions as well as far-reaching consequences for measurement. For example, it is not clear to what extent people can consciously perceive and report on ongoing sub-personal processes. They may experience that a goal is no longer as important to them, but how exactly this importance decreased is usually hard to say and not an intentional act (e.g., from a personal stance, one does not decide to stop loving a person who betrayed them, it “just happens” over time).

By including sub-personal measures in the adapted ostracism-paradigm of Rühs et al. (2022), we simultaneously can address current gaps both in classical ostracism research and in more basic research on goal disengagement processes. Classical research and theories on coping with ostracism (e.g., the *temporal need-threat model of ostracism*, Williams, 2009) so far has not considered sub-personal processes in depth or has at least not been explicit with regard to the personal/sub-personal distinction and the role of intentionality for coping with ostracism. The two-process model, however, is one of the few coping theories that explicitly addresses the importance of sub-personal processes for coping with self-threatening situations, such as ostracism (e.g., Brandtstädter and Rothermund, 2002). Thus, it provides a useful complementary perspective on coping with ostracism.

At the same time, classical ostracism paradigms offer a unique opportunity to study goal disengagement processes experimentally. This has rarely been done in previous research. To be sure, diverse studies have demonstrated the importance of goal adjustment capacities for psychological well-being (for meta-analyses/reviews, see, e.g., Barlow et al., 2020; Brandtstädter and Rothermund, 2002; Heckhausen et al., 2010). However, most of these studies were non-experimental, cross-sectional and used broad indicators of goal adjustment. Thus, we know little about the causal processes underlying goal adjustment. The adapted ostracism paradigm by Rühs et al. (2022) for experimentally studying goal disengagement is a good start to address this. So far, however, only self-report measures for the various facets of goal disengagement have been implemented in it. To address the sub-personal aspects of the two-process model, sub-personal measures need to be included.

Therefore, in the present study, we used the same paradigm as in Rühs et al. (2022) with three indicators of GD: devaluation of the goal as a whole, (relative) devaluation of the ostracizing group, and behavioral deprioritization of members of this group in a subsequent game. But, going beyond the original study, we added two sub-personal indicators of GD: changes in group valence IAT and a Navon task measuring attentional focus. This allowed us to examine possible facets of GD in more detail and from different explanatory stances: self-descriptions, overt behavior, and sub-personal processes. In particular, we examined both the interrelationships among these facets and their relationship to indicators of well-being to address their potential functional role in coping with ostracism. Thus, the present study contributes to a better understanding of GD processes and the development of experimental approaches for its investigation. At the same time, the study provides possible starting points for integrating goal-regulatory aspects into more applied research on coping with ostracism experiences.

### 1.1 Psychological reactions to ostracism—the temporal need-threat model

A popular method for studying ostracism is Williams and Jarvis’ (2006) Cyberball paradigm. In this simple virtual ball game participants are excluded from the game by the other (programmed) participants after a few initial throws. Participants’ reactions to this ostracism experience are compared to those of participants who were included in the game. Much empirical evidence and theoretical development on reactions to ostracism – such as the temporal need-threat model (Wesselmann and Williams, 2017; Williams, 2009; Williams and Nida, 2011) – is based on this and similar experimental paradigms (for a review, see, e.g., Gerber and Wheeler, 2009).

The temporal need-threat model of ostracism postulates three stages of responses to ostracism. The first stage involves *reflexive reactions*. Ostracized individuals are immediately negatively affected by, for example, pain, negative emotions (especially anger and sadness), and threatened fundamental needs. These reactions appear to be relatively universal and insensitive to contextual and individual differences (meta-analysis: Hartgerink et al., 2015; for limitations and restrictions of this assumption, see, e.g., Eck et al., 2016). In the second stage, ostracized individuals show *reflective reactions*. They try to make sense of what has happened to them and use various cognitive (e.g., reappraisal and self-affirmation) and behavioral (e.g., prosocial approach behaviors, aggression, or withdrawal) strategies to cope with the experience and to restore their threatened needs (Eck et al., 2016; Wesselmann et al., 2015). At this stage, contextual and individual factors influence coping responses and the duration of recovery. Prolonged ostracism experiences can exhaust coping resources and lead to a third stage of *resignation*, in which ostracized individuals show severe symptoms, including depression (Riva et al., 2017).

### 1.2 Reactions to ostracism from a goal-regulatory perspective

From a goal-regulatory perspective, an ostracism experience can be described as a situation in which a highly self-relevant goal – the goal to belong to a social group – is blocked (Rühs et al., 2022). Following hierarchical models of motivation (e.g., Elliot and Church, 1997; Kruglanski, 2023), this goal can be considered a concrete manifestation of higher order motivational dispositions, such as the need for self-esteem and the need to belong. Moreover, the need to belong is thought to be an innate basic need (Deci and Ryan, 2000). As the goal to belong to a particular group should be directly linked to it, its blockage should be self-threatening for most people who experience it. This is consistent with findings based on the need-threat model that most people initially react relatively uniformly to ostracism experiences with threatened needs and decreased affective well-being (ostracism as a “strong situation,” see, e.g., Wesselmann and Williams, 2017).

Different developmental regulation theories have pointed out that in goal-blocking situations the ability to flexibly adjust goals, that is, to let go of the goal (*goal disengagement*) and to find and pursue new goals that are consistent with the higher order motivational dispositions (*goal reengagement*), is functional for maintaining a stable self, well-being and agency (e.g., Brandstätter and Herrmann, 2016; Brandtstädter and Rothermund, 2002; Heckhausen et al., 2010; Wrosch et al., 2003a). However, instead of adjusting their goals, ostracized individuals may also stick to the goal to belong to the group and increase investments to overcome the obstacles.

Theories such as the two-process model of developmental regulation (Brandtstädter and Rothermund, 2002) or the motivational theory of lifespan development (Heckhausen et al., 2010) describe the importance of this interplay between goal persistence and goal adjustment for successful developmental regulation. Situational (e.g., action resources, goal substitutability and attainability) and personal (e.g., coping dispositions) factors determine whether, when, and how strongly a person reacts to problems in goal pursuit with persistence or adjustment (e.g., Brandstätter and Bernecker, 2022; Brandtstädter and Rothermund, 2002; Heckhausen et al., 2010; Wrosch et al., 2003b).

In the adapted Cyberball paradigm used in this study, we assume that the temporary goal to belong to a newly formed and anonymous group in an experimental setting is reasonably substitutable, and that the salience of its blockage via ostracism is high enough so that most individuals should respond with goal adjustment in a relatively short time (although interindividual differences in the extent of the adjustment are to be expected, see, e.g., Heckhausen and Wrosch, 2016; Kappes and Greve, 2024).

Various goal regulation theories include different processes under the term goal adjustment (for a recent comparison, see, e.g., Kappes and Greve, 2024). Thus, goal adjustment is better understood as a family of processes rather than a single regulatory process. One central subfamily of goal adjustment processes most theories address (albeit with slightly different conceptualizations) is goal disengagement (GD). Broadly defined, GD involves processes of dissolution of ties to the (blocked) goal that minimize the self-threatening discrepancy between the desired constellation (“ought”) and the actual constellation (“is”). These processes are the focus of the present study.

#### 1.2.1 Goal disengagement processes as coping responses to ostracism

GD can occur cognitively-affectively (i.e., reduced psychological commitment) as well as behaviorally (i.e., reduced effort; Brandstätter and Bernecker, 2022; Wrosch et al., 2003a). Regarding the cognitive-affective facet, different goal regulation theories (e.g., Brandtstädter and Rothermund, 2002; Wrosch et al., 2003a) emphasize these processes of “inner” psychological distancing (e.g., devaluation of the goal, reducing its importance) as particularly important for restoring subjective well-being after goal-blocking experiences. The two-process model of developmental regulation further suggests that this inner distancing is accompanied by a defocalized attention, more holistic processing, greater sensitivity to external stimuli and an increased availability of cognitions that “deconstrue positive goal valences and enhance positive reappraisal of the given situation” (Brandtstädter and Rothermund, 2002, p. 131).

In coping with ostracism, cognitive-affective facets of disengagement from the goal to belong to the (ostracizing) group might be realized in different ways. For example, the global desirability of the concrete goal might decrease in ostracized individuals, the goal is devalued (“It is not so important for me to belong to this specific group anymore”). This could be supported by re-appraisal processes regarding certain sub-aspects of the goal, such as the devaluation of the ostracizing group (“Actually this group is really mean, I do not want to belong to it”). At the same time, attention to environmental stimuli might increase, especially to those that might provide opportunities for engagement in new concrete goals that also serve the more abstract higher-level frustrated needs. In the case of ostracism experiences this could mean that individuals have a broader attentional focus and are more sensitive to social cues and potential opportunities for contact that make a new inclusion experience more likely.

Behavioral facets of disengagement, on the other hand, include reducing investment in goal-directed action, up to and including abandoning goal pursuit altogether and redirecting action resources to new goals. With regard to an ostracism experience, this may manifest itself in reduced approach behavior towards the ostracizing group or avoidance of further contact. Instead, positive interactions with other people should be preferred.

Cognitive-affective and behavioral disengagement do not always go together. For example, individuals sometimes (temporarily) disengage behaviorally from a goal, but that goal remains cognitively-affectively active (“frozen goals,” Davydenko et al., 2019; “goal shelving,” Mayer and Freund, 2022). Most previous research addressing disengagement from a specific goal over time has focused on single facets of disengagement (e.g., cognitive-affective disengagement *or* behavioral disengagement). While valuable insights can already be drawn from these studies regarding particular facets of disengagement, studies that address different facets in their interplay have been rare (for exceptions, see, e.g., Ghassemi et al., 2017; Mayer and Freund, 2022; Vohs et al., 2013). Many questions remain about how the different facets of GD interact, on what time scales they operate, and how they may actually contribute to recovery from a goal blockage experience such as ostracism (for a recent overview of GD research, see Kappes and Schattke, 2022). The present study addresses these questions. We investigate cognitive-affective and behavioral facets of GD in response to an ostracism experience (which implies the blockage of the goal to belong) in a micro-longitudinal experimental design based on Cyberball.

#### 1.2.2 Measuring facets of goal disengagement in response to ostracism

So far, studies investigating facets of GD as response to a concrete goal blockage have mainly used self-report scales for example on goal attainability and desirability or the experience of an action crisis (e.g., Ghassemi et al., 2017), or they have used behavioral measures such as task switching behavior in response to unsolvable tasks (e.g., Kappes and Thomsen, 2020; Koppe and Rothermund, 2017). In the present study, as in Rühs et al. (2022), we combine these different approaches and use self-report measures of cognitive-affective facets of disengagement and a game in which participants’ social actions are observed as a measure of the behavioral facet.

Moreover, in the present study, we complement these “personal” approaches with a sub-personal one. This has rarely been done in studies of GD. Positive exceptions to this (even if they do not refer specifically to GD, but rather to self-regulation in general) include research on affective processing biases and their role for action regulation (for an overview, see, e.g., Rothermund, 2011) and research on automatic self-stabilization (for an overview, see, e.g., Greve and Wentura, 2010). Applying sub-personal approaches specifically to GD could be very informative for several reasons (for a broader discussion, see, e.g., Greve and Wentura, 2007): Some of the GD processes may be less accessible to subjective experience, people may not be fully aware of how they adjust their goals. Moreover, awareness of changes in goal commitment may not even be necessary to benefit from these adjustments concerning subjective well-being (sometimes it may even be detrimental to remember all the things that were once desired but had to be adjusted). Finally, explicit distancing from a goal in self-descriptions may rather reflect wishful thinking (which may itself be a helpful regulatory process in the long run), but it may not necessarily reflect that the person has actually disengaged from the goal from a more sub-personal stance (e.g., regarding implicit associations). In the present study, we address these issues by including sub-personal measures. Especially, participants repeatedly completed an Implicit Association Test (e.g., Pinter and Greenwald, 2011) to measure (possible change in) implicit evaluations of the ostracizing group.^1^

We do not claim that the facets and operationalizations in the present study comprehensively capture GD in response to ostracism. We focus on explicit and implicit indicators of reevaluation processes, as these have been discussed as particularly important for the relieving effect of GD (e.g., Brandstätter and Bernecker, 2022; Brandtstädter and Rothermund, 2002; Wrosch et al., 2003a). These measures, which reflect the cognitive-affective facet of disengagement, are supplemented by an indicator of behavioral disengagement to provide a first insight into the relationship of the facets and their functionality regarding successful recovery after an ostracism experience.

### 1.3 The present research—aims and hypotheses

The study design, hypotheses, and analytical strategy were preregistered on osf.^2^ One goal of the present study was to conceptually replicate the findings of Rühs et al. (2022) that ostracism is an aversive goal blocking experience, and this blockage leads to cognitive-affective and behavioral GD (assumed to be functional in coping with the ostracism experience, however, here, the empirical results were mixed so far). In extension of this, a further contribution of this paper is a closer look at the cognitive-affective facet of GD (especially reevaluation processes) also using sub-personal measures. Moreover, we wanted to test, whether the mixed effects regarding the functionality of GD processes would hold in a replication attempt and also for the new measures. The hypotheses are summarized below (H1 to H10 correspond to the original study to be replicated, hypotheses new in this study refer to sub-personal measures and were numbered consecutively from H11 onwards^3^):

- Manipulation Checks: Positive social interaction in a group and making commonalities salient (*goal induction phase*) lead to an (explicit and implicit) own group bias in all participants (H1). Being excluded (vs. included) in a Cyberball game with the same group afterwards (*goal blockage phase*) should lead to a perceived blockage of the goal to belong to that group (reduced goal attainability, H2) and a decrease in well-being (reduced positive affect and need fulfillment, higher negative affect, H3).
- Facets of GD: If exclusion (vs. inclusion) in Cyberball triggers cognitive-affective facets of GD, after some time for regulation to occur (*goal regulation phase*), participants should report a decrease in subjective desirability of the goal as a whole (H4). Moreover, they should show a reduction in their own group bias, both in explicit and implicit measures (H5, H11), and a broader attentional focus (H12). If behavioral disengagement also occurs, excluded (vs. included) participants in a new social situation should show a behavioral deprioritization of the group members who previously ostracized them (H6).
- Recovery and functionality of facets of GD: After having a few minutes to process the experience (*goal regulation phase*), participants excluded (vs. included) in Cyberball should report a recovery of well-being (i.e., increased positive affect and need fulfillment, decreased negative affect, H7). If facets of GD contribute to this recovery (i.e., if they are functional in this context), then their measures should be associated with these indicators of recovery in excluded participants (H8–H10, H13, H14).

Additionally, we tried to replicate a temporal pattern regarding changes in group evaluation found by Rühs et al. (2022): Excluded participants showed a strong devaluation of their own group directly after exclusion, but this strong devaluation decreased again somewhat as time progressed. Both changes in evaluation (first a decrease and then again an increase in the relative evaluation) were associated with indices of recovery of well-being in the regulation phase and thus seemed to be functional for coping with ostracism.

Going beyond these replications and hypotheses we investigated exploratorily, how personal and sub-personal measures of the different facets of GD were related (both synchronously and asynchronously). We were especially interested whether explicit and implicit measures of group evaluation converged or diverged.

## 2 Materials and methods

Supplementary material is available on osf (e.g., further information on materials, full list of hypotheses and measures, data and codebook, further results, especially regarding the replication and H12, H14).^4^ All studies, measures, manipulations, and data/participant exclusions are reported in the manuscript or its Supplementary material.

### 2.1 Participants

Participation was open to German speakers with access to an Internet-enabled device with a large screen and external keyboard (as the study was conducted online) and were born between 1996 and 2003 (this was important for an interaction task to induce the belonging goal, see below). Persons who studied psychology longer than one semester or had participated in one of the preceding studies with the same paradigm were excluded. Convenience sampling was used, thus the study was advertised through various channels (internet platforms, contacts of study assistants and researchers, educational institutions, flyers in the public).

With respect to the sample size rationale, we followed Rühs et al. (2022). The *a priori* analysis was performed for within-between-interactions in mixed ANOVAs with two groups and three measurement time points because, on the one hand, these contain the statistical tests for the theoretically most relevant hypotheses and, at the same time, required the largest sample size of all tests. To infer within-between interactions in mixed ANOVAs with an effect size of *f* = 0.25 (calculated as in Cohen, 1988), the error probability 𝛼 = 0.05 and a power of 0.85, *N* = 178 participants are needed. Since the present study was designed for groups of six participants at a time, a final *N* of 180 participants was targeted, with some overrecruitment because of foreseeable problems with data quality and internet problems.

A total of *N* = 189 subjects participated in 36 online group sessions from November 2021 to January 2022. Data of one participant was subsequently excluded because of problems in recording the dependent variables in the group session. Therefore, most analyses were conducted with *n* = 188. However, for six participants, the group session data could not be matched to data from a preliminary questionnaire collecting descriptive data and dispositional measures due to ambiguities in the code. These participants were not excluded from hypothesis testing, but the sample description is therefore based on *n* = 182. Most participants (*n* = 145) described themselves as female, 33 as male, and one as diverse (two preferred an individually chosen description, one participant not to answer). The participants were on average 21 years old (*M* = 21.01, *SD* = 1.87) and predominantly students (*n* = 169); some were pupils or trainees (*n* = 9) or were salaried employees or civil servants (*n* = 3, one participant was currently searching for a job).

Participants were randomly assigned to the two conditions of exclusion (*n* = 94) or inclusion (*n* = 94). Between conditions, participants did not differ statistically significantly in age, gender, or employment status. There were also no differences on almost all dispositional control variables (shyness, sociability, need-to-belong, goal disengagement).^5^ However, participants in the inclusion condition had a slightly lower goal reengagement tendency than participants in the exclusion condition, but the effect was small; *M*_inclusion_ = 3.39 (*SD* = 0.67), *M*_exclusion_ = 3.63 (*SD* = 0.59), *t*(180) = −2.51, *p* = 0.01, *d* = −0.37.

### 2.2 Procedure

**Figure 1:**
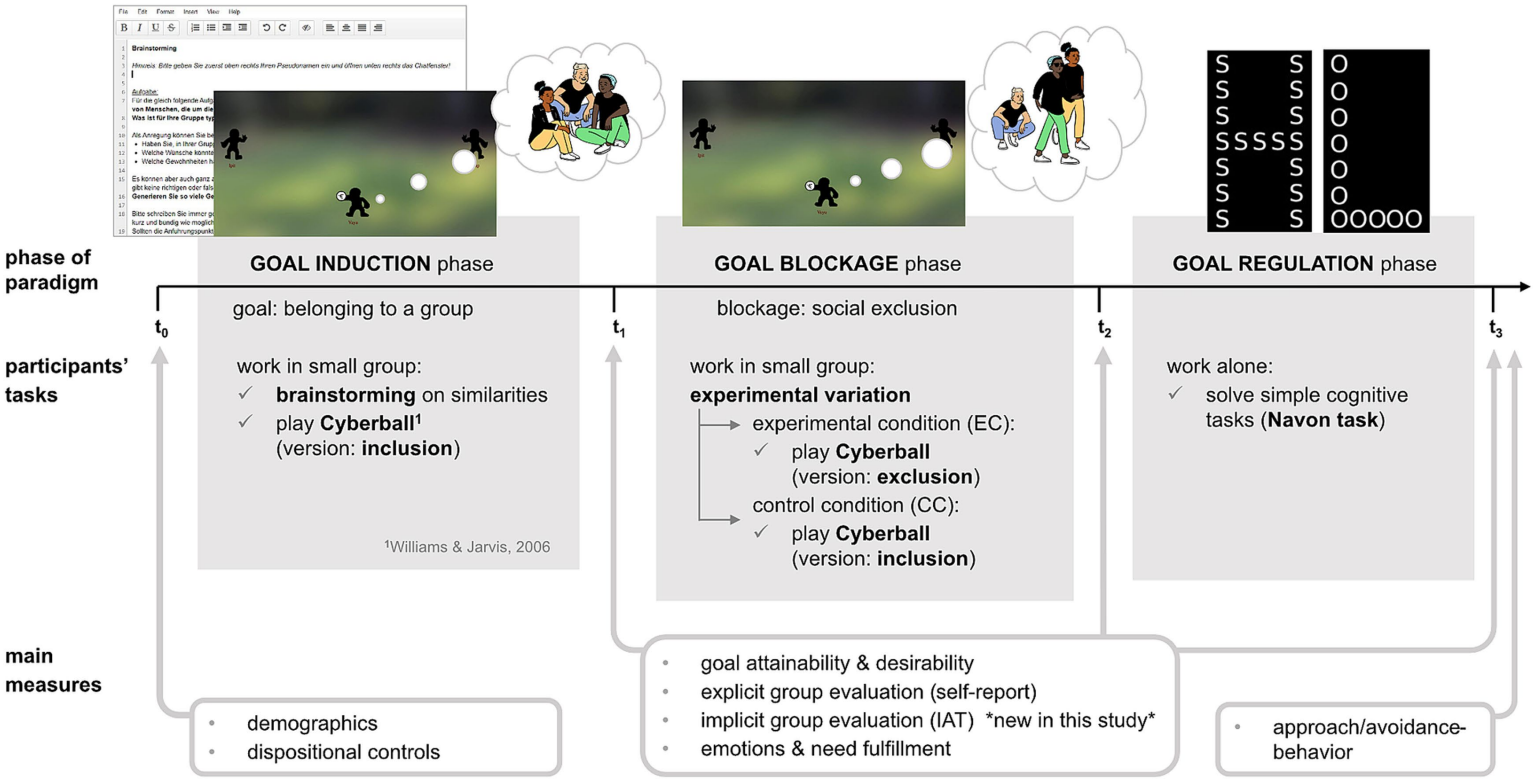
Overview of the experimental procedure. Adapted from “Inducing and blocking the goal to belong in an experimental setting: goal disengagement research using Cyberball” by Rühs et al. (2022). Copyright 2022 by Rühs et al. (adapted with permission).

The overall procedure was the same as in Rühs et al. (2022, see Figure 1). As a cover-story, participants were told the goal of the study was to explore creativity in anonymous online groups. Before they participated in a synchronous online group session, participants filled in a preliminary questionnaire regarding demographics and some dispositional control-variables and provided informed consent (t0).

The online group session was implemented via a video conferencing system. Six participants took part at a time (in 27 times someone did not show up or a place could not be filled, then confederates stepped in). In order to ensure anonymity and to prevent associations with known persons and names, each of them was assigned one of six pseudonyms (Ulut, Ocla, Ydai, Ipit, Delcha, Palup). Cameras and microphones were disabled and participants could not chat with each other, only the general chat for queries to the study supervisor was open. The participants received the instructions via slides and audio and were directed via links to external pages where they worked on the tasks. In the end, participants were fully debriefed and received 15 euros in the form of a voucher (Amazon or Thalia) or a donation to Primaklima e.V. as compensation (psychology students also could choose to earn partial course credit instead).

#### 2.2.1 Tasks in the induction phase

This phase is designed to induce the goal to belong to a group in participants. Participants were divided into two groups of three. They saw the group membership on a slide and had 1 min to learn the pseudonyms of their group members by heart. Then they worked (seemingly) collaboratively on two tasks within this group. First, they did a brainstorming on what they have in common as a group of people born around the turn of the millennium (about 5 min, here the participants actually interacted via chat in a collaborative tool). Second, participants played an inclusive version of Cyberball (seemingly within their group). In Cyberball, a ball game situation is displayed in a simplified way and the player determines by click to whom he wants to pass the ball next. In this study, the virtual teammates were programmed so that every player got the ball equally often (30 throws total, duration about 2 min). The instruction emphasized that it was not about tossing the ball a certain way, but about mentally imagining the whole situation as vividly as possible. This was to make the cover story plausible while also reinforcing the experience.

#### 2.2.2 Tasks in the blockage phase (experimental variation)

This phase is designed to experimentally manipulate the goal blockage experience in participants via social exclusion (goal blockage: experimental condition) or inclusion (no blockage: control condition). Participants played Cyberball seemingly with members of their group a second time. In the control group, participants played the same inclusive version as in the induction phase. Participants in the experimental condition were excluded from the game after two initial throws and had to observe the other two group members passing the ball to each other. It is important to note that participants did not know until the end of the study, whether there would be another opportunity for interaction with the group again. At the beginning, they were informed that they would engage in various individual and group tasks, such as brainstorming, questionnaires, and online ball games, without specifying the exact number or order of tasks. Thus, behaviorally pursuing the goal was blocked in the experimental condition during the game, but it was not clear whether there would be opportunities to pursue the goal again later on.

#### 2.2.3 Tasks in the regulation phase

This phase is designed to give the participants some time for possible regulation processes to unfold. All participants (regardless of condition) worked individually on an implementation of the Navon task (Navon, 1977) designed by Gisbert Stoet for PsyToolkit (Stoet, 2010, 2017). In 50 trials, participants had to identify as fast as possible by keypress, if a stimulus presented on the screen entailed the letter “H” or “O” (target present, key: “c”) or not (no target present, key: “m”). The stimuli were big letters (global feature) composed of small letters (local feature). For the answer of the participant, it did not matter if the “H” or “O” was a global or local feature of the stimulus, it only mattered if it was there at all. Participants had up to four seconds to press a key, otherwise “too slow” was displayed and the next stimulus was presented. For responses within the time period, participants received feedback of their performance (“right” or “wrong”) before the next stimulus was presented.

### 2.3 Measures

After the two tasks of the induction phase (t1), after the second Cyberball game in the blockage phase (t2), and after the Navon task at the end of the regulation phase (t3) dependent variables were measured repeatedly via SoSci Survey (Leiner, 2021) in the following order: goal desirability and attainability, explicit group evaluation, emotions, needs, creativity distractor items, implicit group evaluation. This order and also the operationalization was the same as in Rühs et al. (2022), only the additional measure of implicit group evaluation at the end of each measurement time point was new in the present study. At the end of t3 (after recording implicit group evaluations), participants also played again a version of Cyberball to measure their behavioral approach/avoidance tendency towards members of the own and the other group (baat, there were small changes to the set-up of Rühs et al., 2022, see below). For basic descriptive statistics and indicators of reliability of all repeated variables see Table 1.

**Table 1:** Reliabilities, means, standard deviations, and two-way ANOVA statistics for repeated study variables.

Measure			no blockage		blockage		ANOVA					
	time	α */ r*^†^	*M*	*SD*	*M*	*SD*	Hypothesis	Effect	*F*	*df*	*p*	ω^2^
bga	t1	0.69	55.12	24.20	56.28	22.61		C	7.16	1, 184	0.008	0.02
	t2	0.89	57.88	21.28	42.57	26.03		T	8.80^‡^	1.83, 335.90	<0.001	0.01
	t3	0.87	55.22	20.87	45.29	26.25	H2b	C × T	15.62^‡^	1.83, 335.90	<0.001	0.02
na	t1	0.84	1.55	0.68	1.40	0.55		C	12.83	1, 184	<0.001	0.03
	t2	0.92	1.55	0.74	2.42	1.04		T	31.39^‡^	1.93, 355.66	<0.001	0.06
	t3	0.90	1.62	0.76	1.84	0.84	H3b, H7b	C × T	33.08^‡^	1.93, 355.66	<0.001	0.07
pa	t1	0.81	3.70	0.80	3.69	0.70		C	11.29	1, 184	<0.001	0.03
	t2	0.87	3.55	0.85	2.70	0.76		T	61.57^‡^	1.99, 366.64	<0.001	0.09
	t3	0.86	3.28	0.86	3.16	0.77	H3b, H7b	C × T	33.98^‡^	1.99, 366.64	<0.001	0.05
nf	t1	0.92	3.66	0.63	3.65	0.61		C	62.73	1, 184	<0.001	0.14
	t2	0.97	3.61	0.65	2.22	0.84		T	96.70^‡^	1.81, 332.29	<0.001	0.17
	t3	0.92	3.52	0.58	3.15	0.63	H3b, H7b	C × T	90.15^‡^	1.81, 332.29	<0.001	0.16
bgd	t1	0.80	53.80	24.61	53.17	28.70		C	1.49	1, 184	0.223	0.001
	t2	0.93	56.22	24.64	47.70	31.44		T	14.39	2, 368	<0.001	0.01
	t3	0.96	49.06	27.22	44.17	31.74	H4b	C × T	4.32	2, 368	0.014	0.003
rge	t1	0.87/0.92/0.80/0.90	0.47	0.88	0.43	1.00		C	57.15	1,184	<0.001	0.13
	t2	0.95/0.94/0.89/0.92	0.69	0.97	−1.24	1.86		T	26.90^‡^	1.77, 326.10	<0.001	0.06
	t3	0.94/0.94/0.89/0.93	0.43	0.89	−0.69	1.54	H5b	C × T	40.97^‡^	1.77, 326.10	<0.001	0.09
D_IAT_	t1	0.66	0.54	0.39	0.44	0.36		C	2.94	1, 126	0.089	0.008
	t2	0.47	0.47	0.38	0.41	0.32		T	1.02	2, 252	0.362	<0.001
	t3	0.42	0.49	0.36	0.41	0.30	H11b	C × T	0.13	2, 252	0.876	<0.001

bga = belonging goal attainability; na = negative affect; pa = positive affect; nf = need fulfillment; bgd = belonging goal desirability; rge = relative group evaluation; D_IAT_ = D-Score of the group valence IAT; no blockage = control condition; blockage = experimental condition; C = condition of experimental variation (no blockage vs. blockage); T = time of measurement.^†^For scales consisting of two items (bga and bgd) Pearson’s *r* was used as an estimate of reliability; as rge is a composite score, Cronbach’s α for the four subscales (own-positive, own-negative, other-positive, other-negative) are reported; for the D-Score reliability was estimated following the split-half logic by Pearson’s *r* (corrected by Horst’s Formula) of “D-Scores” calculated separately for the 20-item (block 3 and 6) and 40-item (block 4 and 7) test blocks; for all other scales Cronbach’s *α* is reported.^‡^Mauchly’s test indicated that the assumption of sphericity was violated (*p* < 0.05), Greenhouse–Geisser correction was applied.

#### 2.3.1 Subjective attainability and desirability of the goal to belong to the group (t1–t3)

Regarding two group membership goals (“belong to my group,” “be liked by my group”) participants indicated on a slider how desirable they were to them at the current time in the study and how attainable they seemed to them at that time. Sliders ranged from *not at all important / difficult to attain* to *extremely important / easily attainable* and were recorded as integer numbers from 1 to 101. For the analyses, the arithmetic means both of the attainability and the importance of the two goals were calculated to form the measures belonging goal attainability (bga) and belonging goal desirability (bgd).

#### 2.3.2 Explicit group evaluation (t1–t3)

Participants indicated how much they agreed with 12 evaluative statements. Six statements referred to their own group and six to the other group (three of each were positive, e.g., “I like my group / the other group” and three were negative, e.g., “I reject my group / the other group”). The five-point answer scale ranged from 1 = *not at all* to 5 = *extremely*. For statistical analyses, answers were aggregated into one score of relative group evaluation (rge) using the following formula:


$$\begin{array}{l} rge=\left(M{\left(\mathrm{positive}\right)}_{\mathrm{own}}–M{\left(\mathrm{negative}\right)}_{\mathrm{own}}\right)\\ \;–\left(M{\left(\mathrm{positive}\right)}_{\mathrm{other}}–M{\left(\mathrm{negative}\right)}_{\mathrm{other}}\right)\\ M\left(\mathrm{positive}\right)=\mathrm{arithmetic mean of agreement with positive ratings}.\\ M\left(\mathrm{negative}\right)=\mathrm{arithmetic mean of agreement with negative ratings}.\\ \mathrm{Subscripts own/other refer to the evaluated group}\text{.} \end{array}$$

Positive rge-values indicate a more positive evaluation of the own group compared with the other group.

#### 2.3.3 Emotions and need fulfillment (t1–t3)

Participants rated how strongly they experienced 12 different emotions (six positive: cheerful, happy, relaxed, interested, attentive, determined; six negative: upset, angry, downhearted, sad, afraid, shaky) on a 5-point scale from 1 (*not at all*) to 5 (*extremely*). For the analyses, ratings were averaged for positive and negative emotions separately to get one index representing positive affect (pa) and one representing negative affect (na).

The participants used the same rating scale as for emotions to indicate the extent to which certain needs were met. They rated 20 items of Williams (2009) need-threat-questionnaire translated into German. These items relate to four different superordinate needs (belonging: “I feel I belong to the group”; self-esteem: “I feel liked”; meaningful existence: “I feel useful,” control: “I feel powerful”). However, in this study only one arithmetic mean was calculated across all items (considering item polarity), which was used in the analyses as a composite indicator of the overall degree of need fulfillment (nf).

#### 2.3.4 Implicit group evaluation (t1–t3)

The implicit evaluation of the own group compared with the other group was captured using a standard IAT-protocol of seven blocks by Greenwald et al. (1998) implemented in SoSci Survey (Leiner, 2021). Participants were presented with words stemming from two attitude object categories (“own group”/“other group,” stimuli: three pseudonyms of each group) and two evaluative dimensions (“positive”/“negative,” stimuli: eight positive and eight negative words from the Berlin Affective World List reloaded, Võ et al., 2009). They had to categorize the words as fast as they could by pressing two different keys (left and right). In case of an incorrect response, a red cross appeared, and participants had to correct themselves (reaction time was recorded from stimulus onset until the correct response was given). Time between the correct response and the next stimulus was 250 ms. Between blocks, categories and key assignment rules alternated (for an overview, see Table 2).

**Table 2:** Overview of block specification in the IAT.

Block	Left key	Right key	Function	Trials
1	Own group	Other group	Exercise	20
2	Positive	Negative	Exercise	20
3	Own group + Positive	Other group + Negative	Test	20
4	Own group + Positive	Other group + Negative	Test	40
5	Other group	Own group	Exercise	40^†^
6	Other group + Positive	Own group + Negative	Test	20
7	Other group + Positive	Own group + Negative	Test	40

The D-Score of association strength was calculated according the improved scoring algorithm by Greenwald et al. (2003), using reaction times from block 3, 4, 6, and 7.^†^as recommended by Nosek et al. (2005) 40 instead of 20 trails were used in block 5 to minimize sequence effects.

Evaluation of reaction times followed the improved algorithm of Greenwald et al. (2003) for calculating a D-Score that represents the relative implicit evaluation of the own group compared with the other group. In summary, the D-Score compares standardized average reaction times of the blocks with different key assignments. In the present study, reaction times from blocks 3 and 4 (own + positive / other + negative) were subtracted from reaction times in blocks 6 and 7 (own + negative / other + positive). Reaction times should be shorter in blocks where key assignments align more with the object-evaluation associations (e. g. in blocks 3 and 4 if an own group bias is present). Generally speaking, the stronger the positive associations and the weaker the negative associations of the own group were in relation to those of the other group, the higher the D-Score (positive D values speak in favor of an implicit preference of the own group).

#### 2.3.5 Behavioral approach/avoidance tendency (t3)

After the IAT at t3, participants played an inclusive version of Cyberball again (every player got the ball equally often). However, this time the two ostensible co-players were mixed from both groups (one player from the own group and one from the other group). The first throw was always from a co-player to the participant to have an inclusive start (group membership of the first thrower was counterbalanced within conditions). Deviating from Rühs et al. (2022), the game lasted 45 (instead of 30) throws so that participants had more throws to distribute to the other two players (depending on the course of the game, the participant could make 14 to 16 throws). Each throw, the participant could decide whether to pass the ball to a member of its own or the other group. To have an index of behavioral approach/avoidance tendency (baat), the proportion of throws to the member of the other group out of the total number of throws by the participant was calculated. Values above 0.5 represent a preference for the player of the other group.

### 2.4 Analysis plan

All confirmatory analyses were preregistered. In the presentation of the results, the hypotheses to which the respective analyses refer are indicated. Only the analytical procedure with regard to the repeated measurement variables will be briefly outlined here, since two different methods were used in parallel.

First, differences between experimental conditions in intraindividual changes between measurement time points were analyzed. For this purpose, two change variables (t1 to t2: Δt_12_ and t2 to t3: Δt_23_) were calculated for each repeated measurement variable. The earlier measurement timepoint was always subtracted from the later one so that positive change values correspond to an increase and negative change values to a decrease over the two measurements. Deviating from Rühs et al. (2022) a third change-score was also calculated from t1 to t3 for explicit and implicit group evaluation measures (Δt_13_). Rühs et al. (2022) provided initial indications for an excessive devaluation of the own group at t2 and a subsequent slight reevaluation at t3. Even if this nonlinear course is interesting in detail (and should be replicated in this study), the absolute difference of the evaluation between t1 and t3 could also be important regarding the assumed relieving effect (functionality) of the reevaluation processes. For testing the different hypotheses, average change scores were compared between the experimental and control condition using one-sided independent *t* tests (all *t* tests and their correspondence to the hypotheses are displayed in Table 3).

**Table 3:** Means, standard deviations, and *t-*test statistics for change variables, and behavioral approach/avoidance tendency.

Measure	No blockage		Blockage		*t* test				
	*M*	*SD*	*M*	*SD*	Hypothesis	*t*	*df*	*p*	*d*
bga Δt_12_	2.76	16.78	−14.66	26.66	H2a (𝜇_nb_ > 𝜇_b_)^†^	5.36	186	<0.001	0.78
bga Δt_23_	−2.66	14.57	2.72	19.25	add. (𝜇_nb_ ≠ 𝜇_b_)	−2.15	184	0.033^§^	−0.32
na Δt_12_	−0.01	0.74	1.01	1.11	H3a (𝜇_nb_ < 𝜇_b_)^†^	−7.41	186	<0.001	−1.08
na Δt_23_	0.07	0.68	−0.58	0.93	H7a (𝜇_nb_ > 𝜇_b_)^†^	5.48	184	<0.001	0.80
pa Δt_12_	−0.15	0.75	−1.00	0.78	H3a (𝜇_nb_ > 𝜇_b_)	7.61	186	<0.001	1.10
pa Δt_23_	−0.27	0.68	0.46	0.78	H7a (𝜇_nb_ < 𝜇_b_)^‡^	−6.79	184	<0.001	−1.00
nf Δt_12_	−0.05	0.66	−1.46	1.00	H3a (𝜇_nb_ > 𝜇_b_)^†^	11.44	186	<0.001	1.67
nf Δt_23_	−0.09	0.60	0.93	0.77	H7a (𝜇_nb_ < 𝜇_b_)	−10.04	184	<0.001	−1.47
bgd Δt_12_	2.43	13.93	−4.86	22.51	add. (𝜇_nb_ ≠ 𝜇_b_)	2.67	186	0.008	0.39
bgd Δt_23_	−7.17	17.03	−3.53	17.93	H4a (𝜇_nb_ > 𝜇_b_)	−1.42	184	− ^¶^	−
rge Δt_12_	0.22	0.98	−1.69	2.06	add. (𝜇_nb_ ≠ 𝜇_b_)^†^	8.11	186	<0.001	1.18
rge Δt_23_	−0.26	0.82	0.55	1.44	add. (𝜇_nb_ ≠ 𝜇_b_)^†^	−4.75	184	<0.001	−0.70
rge Δt_13_	−0.04	0.89	−1.12	1.89	H5a (𝜇_nb_ > 𝜇_b_)^†^	5.01	184	<0.001	0.73
D_IAT_ Δt_12_	−0.06	0.46	−0.05	0.36	add. (𝜇_nb_ ≠ 𝜇_b_)	−0.15	131	0.884	−0.03
D_IAT_ Δt_23_	0.02	0.40	0.00	0.33	add. (𝜇_nb_ ≠ 𝜇_b_)	0.32	127	0.753	0.06
D_IAT_ Δt_13_	−0.03	0.44	−0.03	0.37	H11a (𝜇_nb_ > 𝜇_b_)	−0.04	130	0.515	−0.01
baat	0.51	0.12	0.55	0.16	H6 (𝜇_nb_ < 𝜇_b_)^‡^	−2.23	176	0.013	−0.34

bga = belonging goal attainability; bgd = belonging goal desirability; na = negative affect; pa = positive affect; nf = need fulfillment; rge = relative group evaluation; baat = behavioral approach/avoidance tendency; no blockage = control condition; blockage = experimental condition.^†^Shapiro–Wilk test suggested deviation from normality and Levene’s test non equal variances; however more robust Mann–Whitney and Welch test yielded same results regarding decision for rejecting the null-hypothesis, thus statistics for Student *t* test are reported.^‡^Shapiro–Wilk test suggested deviation from normality; however more robust Mann–Whitney test yielded same results regarding decision for rejecting the null-hypothesis, thus statistics for Student *t* test are reported. ^§^Shapiro–Wilk test suggested deviation from normality; more robust Mann–Whitney test yielded non-significant results (*U* = 3764.5, *p* = 0.128).^¶^One-sided significance test was not performed because mean difference was in the opposite direction as hypothesized.

**Figure 2:**
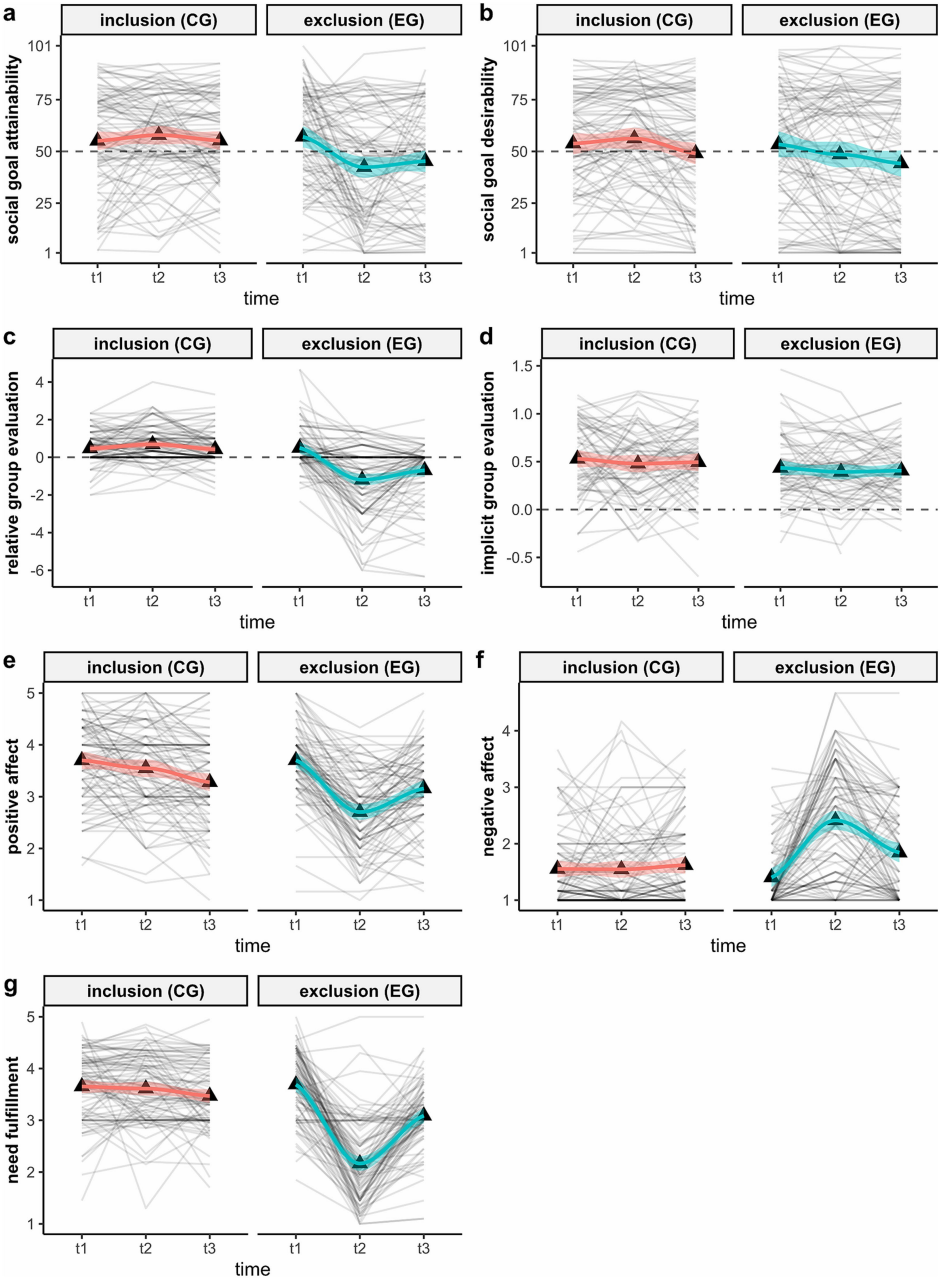
Trajectories of repeatedly measured variables from t1 to t3 [**(a)** = social goal attainability; **(b)** = social goal desirability; **(c)** = relative group evaluation; **(d)** = implicit group evaluation; **(e)** = positive affect; **(f)** = negative affect; **(g)** = need fulfillment] depending on the experience of ostracism between t1 and t2. CG = control condition (no blockage); EG = experimental condition (blockage); gray lines = trajectories of individual participants; colored lines = smoothed trajectories (Loess method) with 95% CI; triangles = mean values across individuals within conditions.

Second, two-factorial ANOVAs with repeated measures on one factor (time of measurement: t1, t2, t3) were performed for all variables measured three times (all ANOVAs and their correspondence to the hypotheses are displayed in Table 1). For hypotheses testing, primarily the interaction effect of condition × measurement time point was interpreted. In addition, planned contrasts were calculated to test specific assumptions of the hypotheses. For each repeated variable, these were a total of 4 contrasts: one contrast to compare the two conditions at each measurement time point (t1: C1, t2: C2, t3: C3) and one contrast specifying whether the condition differences differed in magnitude between t2 and t3 (C4) to specify the interaction effect (detailed results can be found in the Supplementary material on osf). For an impression of interindividual differences within experimental conditions, individual trajectories over time were plotted using R (Figure 2).

All analyses were performed using JASP 0.16.3 (JASP Team, 2022). Results were considered to be statistically significant when *p* < 0.05.^6^

## 3 Results

One goal of the present study was to conceptually replicate the findings of Rühs et al. (2022). A full report of the replication results is beyond the scope of this paper, a compilation can be found as Supplementary material on osf.

### 3.1 Manipulation checks: goal induction and goal blockage (H1–H3)

As assumed in H1, after the induction phase (t1), all participants (regardless of experimental condition) rated their own group more positive than the other group; *M*_own_t1_ = 1.88 (1.39), *M*_other_t1_ = 1.40 (1.10), *t*(187) = 6.66, *p* < 0.001, *d* = 0.49. This own group bias was also obtained in the implicit IAT measure: the mean D-Score over all participants at t1 was positive (*M*_D_t1_ = 0.48, *S*_D_t1_ = 0.38) and was statistically significantly greater than zero; *t*(138) = 15.02, *p* < 0.001. Moreover, mean desirability and attainability ratings of the goal to belong to the group were slightly above the scale mean for all participants at t1 (see also Table 1).

During the blockage phase (from t1 to t2), subjective goal attainability decreased in participants who were excluded in Cyberball (and stayed low from t2 to t3) whereas participants who were included reported no change (H2, see Table 3 for *t-*tests comparing the change variables, Table 1 for the ANOVA approach and Supplementary Table 1 on osf for the corresponding calculated contrasts). Furthermore, excluded (compared with included) participants reported a decrease in well-being (reduced positive affect and need fulfillment, higher negative affect) during the blockage phase from t1 to t2 (H3, see Tables 1, 3 and Supplementary Table 1).

### 3.2 Facets of goal disengagement (H4–H6, H11)

**Figure 3:**
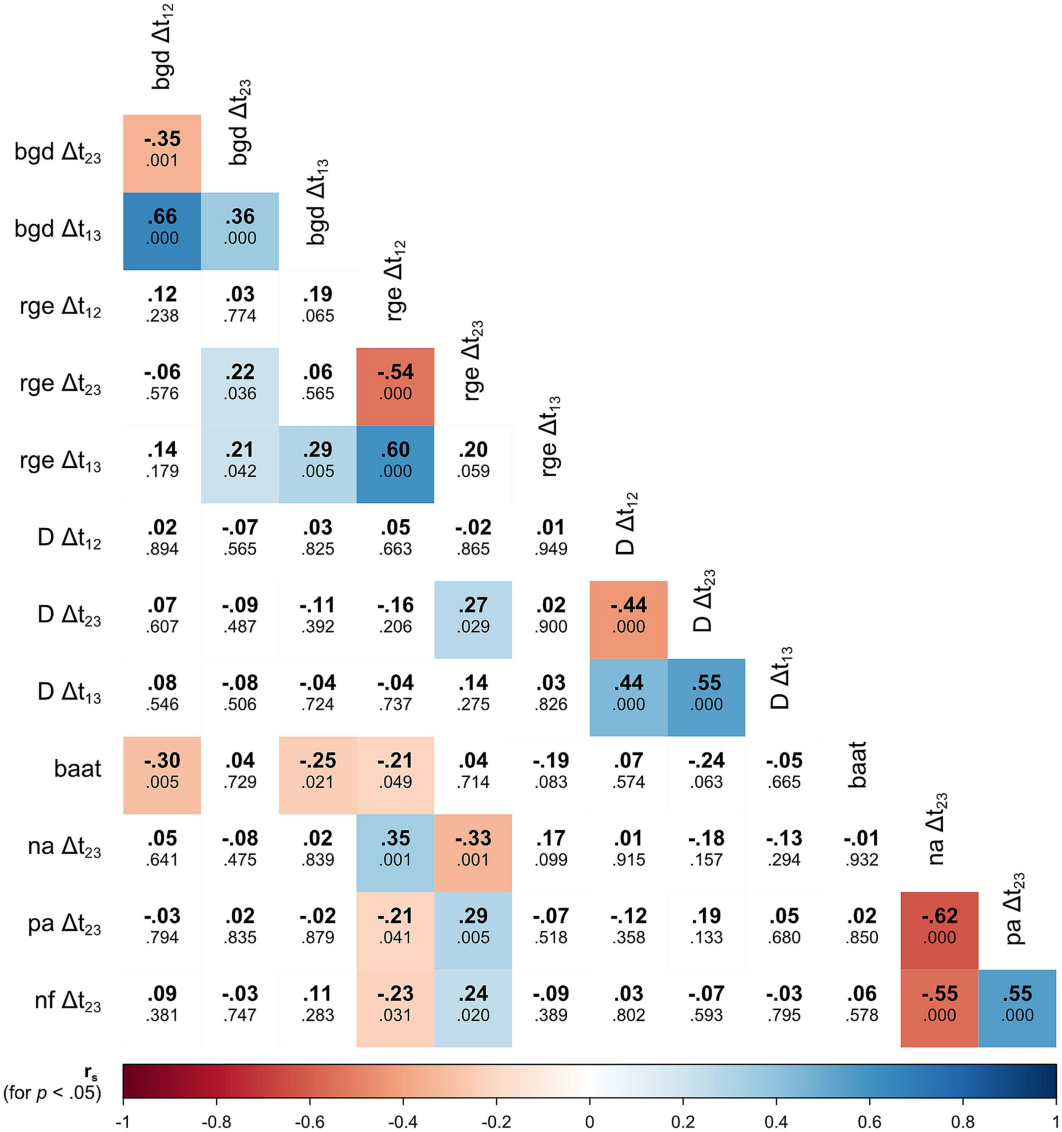
Heat map of bivariate correlations between measures of recovery in the regulation phase and facets of goal disengagement (exclusion condition). Bold numbers represent Spearman’s rank correlation coefficients, smaller numbers below represent *p* values of the corresponding test. Statistically significant results (*p* < 0.05) are highlighted (color scale proportional to correlational strength). *n* = 64–94 (pairwise exclusion, especially in the IAT data of some participants did not met criteria to calculate the D-Score). bgd = belonging goal desirability; rge = relative group evaluation; D = D-Score of the group valence IAT; na = negative affect; pa = positive affect; nf = need fulfillment; baat = behavioral approach/avoidance tendency. Data for the control condition can be found in Supplementary Table 2 on osf.

H4 to H6 and H11 entailed assumptions about possible cognitive-affective (reevaluation processes regarding the goal and the group) and behavioral facets (behavioral approach/avoidance tendency in a social situation) of GD that should be triggered by an ostracism experience. All results regarding these hypotheses are displayed in Tables 1–3 (and Supplementary Table 1) and Figures 2, 3 in detail. In the following, we provide an overview of the main findings and their correspondence to the hypotheses.

#### 3.2.1 Devaluation of the goal as a whole (H4)

As assumed in H4, participants who got excluded reported a decrease in subjective goal desirability, both from t1 to t2 and from t2 to t3. The decrease from t1 to t2 in the experimental condition was also statistically significantly more pronounced than that of the control condition (that showed no decrease during that time, Table 3). Moreover, the ANOVA showed a statistically significant condition × time interaction effect of small size (Table 1 and Supplementary Table 1). However, from t2 to t3, participants in the control condition unexpectedly also reported a decrease in goal desirability, that even was more pronounced than that in the experimental condition during that time. Thus, goal desirability change in the regulation phase differed between conditions in the opposite direction than assumed for the one-sided *t*-test (it was more negative for the control group), so this test was not performed (Table 3).

#### 3.2.2 Explicit (H5) and implicit devaluation (H11) of the group

Regarding group reevaluation processes, participants in the exclusion (compared with inclusion) condition showed an overall reduction of the own group bias from t1 to t3 in the explicit measure (H5, Tables 1, 3 and Supplementary Table 1). Moreover, also the expected pattern of change over time could be found in excluded participants: There was an initial reversal of the explicit own group bias to a preference of the other group in the blockage phase (t1–t2). During the regulation phase from t2 to t3 this preference of the other group reduced somewhat but remained.

Against expectations, no changes over time could be found in the implicit group evaluation measure (IAT) in both conditions (H11, Tables 1, 3 and Supplementary Table 1). Participants in both conditions showed positive D-Scores that did not differ between conditions at all measurement time points, indicating a constant implicit own group bias.

#### 3.2.3 Behavioral deprioritization of own group members (H6)

At the end of the regulation phase (after the last IAT) excluded participants showed behavioral disengagement as reflected in a behavioral deprioritization of own group members (H6). While included participants distributed the balls evenly to both teammates in the last Cyberball game with mixed coplayers (*M*_baat_ = 0.51), excluded participants showed a behavioral approach/avoidance tendency in favor of the player of the other group (*M*_baat_ = 0.55, for detailed results see Table 3). We additionally investigated (exploratively) to whom participants threw their first ball depending on experimental condition. Here, the difference in preference was even more visible. While only 27 out of 87 excluded participants made the first throw to their own group member, 46 of 90 included participants did so; Cramer’s *V* = 0.20, *x*^2^ = 7.36, *p* = 0.007.

### 3.3 Relation between different facets and temporal patterns (exploratory)

To see how the different facets of GD were related in excluded participants, we exploratorily examined bivariate correlations of change measures for repeated variables (goal desirability, explicit and implicit group evaluation) and the behavioral approach/avoidance tendency (Figure 3). Some statistically significant correlations with smaller effect sizes were found.

Behavioral approach/avoidance tendency at t3 correlated negatively with several prior reevaluation processes. That is, a stronger decrease in goal desirability from t1 to t2 (and from t1 to t3) and a stronger decrease in the explicit own group bias from t1 to t2 were both predictive of a higher preference of the player of the other group in the Cyberball game at t3 (a higher baat-score indicates a deprioritization of the own group). Moreover, changes in goal desirability from t2 to t3 and from t1 to t3 were associated with simultaneous changes in explicit group evaluation in the same direction. Interestingly, from t2 to t3, measures of change in explicit and implicit group evaluation were slightly positively correlated, although the overall pattern of results on explicit and implicit group evaluations was otherwise very different (as there were strong effects of the experimental manipulation on explicit, but not on implicit measures).

Another interesting pattern emerged when the time-lagged correlations of indicators of changes in evaluations were considered. For goal desirability as well as for explicit and implicit group evaluation, the change from t1 to t2 was negatively associated with the change in the same variable from t2 to t3. This suggests a non-linear trend, the assumption of which seems plausible when looking at the trajectories over time of participants individually (Figure 2).

### 3.4 Recovery and functionality of facets of goal disengagement (H7–H10, H13)

During the regulation phase, participants in the exclusion condition (compared with those in the inclusion condition) reported a recovery of well-being indicated by an increase in positive affect and need fulfillment and a decrease in negative affect from t2 to t3 (H7, Tables 1, 3). This recovery was nearly complete; differences between experimental and control conditions at t3 were statistically non-significant for positive and negative affect and significant but rather small for need fulfillment (see Supplementary Table 1 for details of calculated contrasts).

It was assumed, that the different facets of GD contribute to recovery of well-being after ostracism (i.e., that they are functional in this context). Thus, their measures should be associated with an increase in need fulfillment and positive affect as well as with a decrease in negative affect from t2 to t3 for excluded participants (H8–H10, H13). However, almost no such correlations could be found. Only change in explicit group evaluation was associated with recovery indicators in a complex way: Change in explicit group evaluations from t1 to t2 positively predicted change in negative affect from t2 to t3 and negatively predicted change in positive affect and need fulfillment from t2 to t3. Thus, if participants devalued their group from t1 to t2, they experienced a restoration of well-being afterwards from t2 to t3. Interestingly, for explicit group evaluation change from t2 to t3, this association pattern was reversed: synchronously, change in explicit group evaluation was negatively associated with change in negative affect and positively associated with change in positive affect and need fulfillment. If participants’ evaluation of the own group improved in the regulation phase from t2 to t3, their well-being synchronously improved. The index for the net amount of change in explicit group evaluation from t1 to t3 was not associated with indicators of recovery from t2 to t3. Neither were all other indicators of facets of GD (change in goal desirability, change in implicit group evaluation, behavioral approach/avoidance tendency).

## 4 Discussion

### 4.1 Conceptual replication

One aim of the present study was to conceptually replicate the findings of Rühs et al. (2022). In sum, almost all findings of Rühs et al. (2022) could be replicated. Consistent with the original study, being excluded (compared with being included) in Cyberball led to a blockage of the goal to belong (lower goal attainability) and deterioration of well-being (lower need fulfillment and positive affect, higher negative affect) immediately after the game. These findings are consistent with the large body of research demonstrating the detrimental and self-threatening effects of ostracism on individuals (for a compilation, see, Williams and Nida, 2017). Furthermore, as in the original study, the present results extend these findings by empirically demonstrating that current goal pursuit is also affected by ostracism experiences. The present study replicated the finding that goal blockage through ostracism leads to processes associated with GD, such as devaluation of the belonging goal and the ostracizing group, as well as behavioral deprioritization of members of this group in a subsequent social game. These results fit well with theories and empirical findings that postulate a causal relationship between goal blockage and goal adjustment processes and especially emphasize reevaluation processes in this context (e.g., Brandtstädter and Rothermund, 2002; Heckhausen et al., 2010).

As in other studies using the Cyberball paradigm with repeated measures (e.g., Clear et al., 2021; Molet et al., 2013; Zadro et al., 2006), participants in the present study largely recovered from exclusion within a short period of time. However, the recovery of well-being from t2 to t3 was not statistically significantly associated with the various indicators of GD used, with the exception of the particular pattern of changes in explicit group evaluations (devaluation from t1 to t2 and some neutralization from t2 to t3 were associated with recovery of well-being from t2 to t3). This is largely consistent with the findings of Rühs et al. (2022), except that they additionally found a statistically significant negative correlation of small effect between the change in goal desirability from t2 to t3 and the change in negative affect during this time. This effect could not be replicated in the present study (see below for a more detailed discussion of the ambiguous findings regarding the functionality of GD).

### 4.2 Facets of GD—personal and sub-personal approaches, correlations and temporal patterns

Beyond replication, this study’s particular focus was a closer look at cognitive-affective facets (especially reevaluation processes) and behavioral facets of GD, their measurement, their interrelationships and their role in coping with ostracism. Although some of the measures still need to be refined, the present study has shown that it is possible and worthwhile to address different facets of situational GD in one design. Even though precise assumptions about temporal relationships are still lacking (it is not even certain that the different postulated processes are synchronous in time), the present study was able to find some associations between the various indicators of GD used. For instance, a devaluation of the goal to belong and an explicit devaluation of one’s own group immediately after the exclusion experience (cognitive-affective disengagement) were predictive of a deprioritization of the ostracizing teammate in the last mixed Cyberball game (behavioral disengagement). This highlights that devaluative effects are unique to the ostracizing group member and associated with particular behavioral reactions, rather than being merely a result of general decreased motivation.

Regarding the applied sub-personal measures of facets of GD (changes in implicit group evaluations and the Navon task) we only found one association of rather small effect size with the more “personal” measures of GD: changes in implicit evaluations from t2 to t3 were positively associated with changes in explicit evaluations over the same period. Overall, however, no group-level effect of ostracism on implicit group evaluations was found. However, since the reliability of the IAT was not optimal, implicit group evaluations should be interpreted with some caution. Nevertheless, a stable own group bias was found in both experimental groups, which is consistent with standard findings in social psychological research, even for minimal groups (e.g., Ashburn-Nardo et al., 2001). This provides some validation of the measure.

Given the general assumption that belonging to a positively valued group is experienced as rewarding and serves a positive social identity (Tajfel and Turner, 1979), devaluing that group when ostracized from it may buffer the negative effects of the obstacle to achieving the goal to belong to this group. The missing effect of the experimental manipulation on implicit group evaluations is interesting because the effect on explicit group evaluations was strong. This divergence between implicit and explicit measures is also repeatedly reported in other studies comparing implicit and explicit attitude change (see, e.g., Gawronski and Bodenhausen, 2006, for a review and theoretical account, or Payne et al., 2008, for a study investigating the role of structural fit of implicit and explicit measures). One possible explanation could be that the IAT is not sensitive enough at the individual level to detect the relevant (micro-)changes in social-cognitive processing (e.g., Connor and Evers, 2020; Cummins and Hussey, 2023). Thus, further studies using different implicit and repeated measures are needed to address (change) sensitivity. However, if it is not a measurement problem, the question arises as to what it means for the hypothesized processes of GD if a person devalues his or her group in self-report (i.e., appears to disengage cognitively-affectively at least somewhat), but implicitly still has strong cognitive associations of the group with positive attributes. Did this person disengage or not? Or only partially?

Helpful insights might be drawn from recent research on habitual behavior and so-called “action slips” (e.g., Buabang et al., 2023a; Buabang et al., 2023b). These studies have shown that individuals can change previously established (habitual) behavior in experimental settings (e.g., because rewards for certain behaviors change within the experiment): Individuals understood that behavioral values had changed, and old contingencies in guiding behavioral responses could be “overridden” if sufficient cognitive resources were available. However, especially in stressful situations, old contingencies could “resurface” and guide behavior again. Applied to the divergence of explicit and implicit changes in group evaluations after the ostracism experience in the present study, this could mean that the devaluation of one’s own group measured by explicit measures is indicative of newly learned contingencies and new mental representations (e.g., own group - negative) and thus of goal disengagement. The fact that this is not reflected by the implicit measure could be due to the high time pressure in the IAT task, which leads to a stronger influence of older (still existing) contingencies (e.g., own group - positive). Finally, the question arises as to which processes contribute to attitude change, and how these (re-)learning processes are expressed in the various implicit and explicit measures (e.g., Corneille and Stahl, 2019; Gawronski and Bodenhausen, 2006). Very informative in this regard could be findings from research on evaluative conditioning, that delve deeper into possible learning processes (e.g., associative and propositional processes) of stimulus-valence associations and factors influencing them (e.g., De Houwer, 2018; Hu et al., 2017; Reichmann and Hütter, 2024; for a recent review, see Moran et al., 2023). Future research could apply these findings to GD processes. In doing so, an interesting question could also be what role personal intentions to disengage play in the occurrence of sub-personal processes. Theoretical considerations in the context of the two-process model (e.g., Brandtstädter and Rothermund, 2002) as well as empirical evidence from evaluative conditioning paradigms (e.g., Gawronski et al., 2014; Gast and Rothermund, 2011) suggest that such intentions and personal reactions may indeed have a facilitating effect on disengagement. Taken together, although linking the two stances remains challenging (e.g., Greve and Wentura, 2007; Rothermund et al., 2020), adding more research from a sub-personal stance (investigating underlying mechanisms) to the research that has so far been primarily conducted from a personal stance, might be a fruitful avenue for a better conceptual clarification and understanding of GD processes.

Unfortunately, the second sub-personal measure in the present study was not very informative in this regard. Results suggested that the implementation of the Navon task in the current design was not sufficiently reliable to be a sensitive measure of attentional focus in this context. Future studies should use settings with greater standardization possibilities (laboratories) and different task implementations to examine whether differences in attentional focus can be found between included and excluded participants.

Lastly, even though stable experimental effects could be found at the group level for most dependent variables (except the IAT and the Navon task), there seems to be some variance in individual trajectories (see Figure 2). It was particularly striking that (at least by eye) all variables representing evaluations (of the goal or the group: bgd, rge, D) did not show a linear development over time at the individual level, but rather u-shaped progressions. This was also indicated by the negative correlations between the change measures from t1 to t2 and from t2 to t3 for these three variables. For future research, this again illustrates the relevance of (a) hitting the “right” time for certain inquiries, as the processes could run in completely different directions at different points in time and (b) also to look beyond group level analyses at individual (not only linear) time trajectories.

### 4.3 Are GD processes functional for coping with self-threatening goal blockage situations such as ostracism?

This study focused on GD processes because developmental self-regulation theories (e.g., Brandtstädter and Rothermund, 2002; Heckhausen et al., 2010) discuss these processes as particularly significant for maintaining well-being in goal blockage situations. While the study clearly demonstrated that ostracism, as a goal-blocking situation, triggers some of the core processes hypothesized in these theories (such as reevaluations and behavioral adjustments), the findings on whether these processes can help to restore well-being are mixed. We were able to replicate the pattern by Rühs et al. (2022) that both an initial strong devaluation of the own group in the self-report (from t1 to t2) and a subsequent relativization of this devaluation and slight upgrading of the group (from t2 to t3) were associated with a recovery of well-being a few minutes after ostracism (regarding change in need fulfillment, positive and negative affect from t2 to t3). However, absolute devaluation from t1 to t3 was not associated with recovery of well-being. This calls for more differentiation of goal regulation theories, which in particular speak of reducing so-called is-ought discrepancies (e.g., by devaluing partial aspects of the goal or the goal as a whole). Apparently, it is not the mere difference between the prior and the posterior evaluation that is important, but also the time course of the evaluation to get to this point.

Moreover, with respect to the other indicators of facets of GD (decrease in goal desirability and behavioral deprioritization), no associations with restoration of well-being were found either. This is despite the fact that downgrading of the blocked goal is assumed to be a central mechanism of the relieving effect of goal adjustment brought up by the theories addressed (for a review, see also Brandstätter and Bernecker, 2022). How should the missing associations in this study be interpreted? There is some evidence, that the missing associations may in fact be “real” rather than a methodological or sampling effect. While we have previously assumed that the subfamily of GD processes plays an important role for the “relieving effect” of goal adjustment in goal blocking situations, some recent studies call this into question (e.g., Barlow et al., 2020; Kappes and Greve, 2024; Loidl and Leipold, 2019). These studies differentiate the broad family of goal adjustment processes into subfamilies of processes and assess their differential associations with indicators of well-being. Although the specific research concerns of these studies vary, they share an interesting pattern of findings: Measures that primarily target some kind of “letting go” of the blocked goal (e.g., “goal disengagement” in Barlow et al. (2020), and in Kappes and Greve (2024); “goal detachment” in Loidl and Leipold (2019) showed only weak or even negative associations with measures of well-being. In contrast, broader measures involving different sets of processes (e.g., “accommodative coping” or “compensatory secondary control,” Kappes and Greve, 2024) or measures addressing other individual aspects of goal adjustment, such as some kind of reorientation (e.g., Barlow et al., 2020; Kappes and Greve, 2024; Loidl and Leipold, 2019) or specific reappraisal processes (Loidl and Leipold, 2019), were largely positively associated with indicators of well-being. Although the employed measures of “letting go” in these studies are mainly dispositional and sometimes difficult to distinguish from (negatively connoted) resignation and thus differ from the operationalization of GD in this study (which is situational and mainly related to reevaluation processes), they share the focus on the dissolution of ties to the (blocked) goal. Future studies could combine the approach of experimentally varying regulation strategies, which has already been done (e.g., Hales et al., 2016; Molet et al., 2013), with investigating their differential effects on personal and sub-personal processes.

Taken together, the results suggest that simply loosening the ties to a blocked goal (GD in this study, whether cognitive-affective or behavioral makes no difference in the present study) is not sufficient for goal adjustment to be relieving and self-stabilizing in goal-blocking situations. What exactly causes its mitigating effect, is not yet well understood. Goal adjustment processes (and GD in particular) need empirically informed conceptual sharpening, especially with regard to their potential functional role in coping with self-threatening situations, such as ostracism. One possibility for future studies could be to test the paradigm used in this study with other dependent variables (for the different facets of goal adjustment). In addition, specific experimental manipulations of the various goal-adjustment processes could be introduced to directly address their causal influences on well-being and self-stability in goal-blocking situations.

### 4.4 Implications for research on coping with ostracism

There is an ongoing debate about the consequences of ostracism on interpersonal responses, as the full range of prosociality to aggression to solitude seeking has been observed in victims (e.g., Maner et al., 2007; Ren et al., 2016; Twenge et al., 2007; Wesselmann et al., 2015). The present study found this high variety even in a single design (i.e., in a highly standardized setting). Victims’ attitudes toward the ostracizing group were measured using different approaches: explicit group evaluation (self-report), implicit group evaluation (IAT), and approach/avoidance behavior towards ostracizing individuals compared to new individuals (last Cyberball game). For ostracized individuals, both devaluation processes (from t1 to t2) and positive reevaluation processes (from t2 to t3) toward the ostracizing group were observed at the group level. Regarding implicit associations, there was no change in relative group evaluations at the group level. Behaviorally, ostracized individuals on average deprioritized interactions with the former ostracizing group members relative to interactions with new players. In addition, measures of (explicit and implicit) evaluation (change) showed high interindividual variance. A more detailed analysis (considering temporal aspects) of the different measures of attitudes towards the ostracizing group could help to differentiate and test hypotheses regarding potential moderating factors of the relationship between ostracism and interpersonal reactions to it.

Also, with respect to the course of the classic outcome variables of ostracism research (need-fulfillment, positive and negative affect), substantial interindividual variance was found even for the change from t1 to t2 (i.e., the change from before to immediately after the ostracism experience, reflexive reactions). This somewhat contradicts the need-threat model’s assumption that ostracism is a relatively strong situation and that responses should be fairly uniform in the reflexive phase, whereas they should vary more across individuals in the reflective phase (e.g., Wesselmann and Williams, 2017). However, there is a lack of precise information about the time frames assumed for the reflexive and reflective phases in the need-threat model (and how much variation would be acceptable to speak of a uniform response). Repeated measures designs such as the one in the present study could help to specify and test these assumptions in future studies.

Finally, this study showed that, in addition to the more commonly studied effects (on needs, affect, and pro−/antisocial tendencies), ostracism also causes problems at the level of goal pursuit. The goal-regulatory processes involved open up a new perspective from which coping with ostracism could be explored and possibly (in the long run) interventions could be developed.

### 4.5 Generalizability of results

Regarding the generalizability of the results, it should be noted that the study used a mostly student, mostly female sample from a WEIRD (Henrich et al., 2010) society. Furthermore, we used a highly controlled experimental setting to test our hypotheses, so the external validity can be questioned. Is ostracism in Cyberball comparable to ostracism in real-life? And is this kind of goal blocking comparable to real-life goal blocking situations? Both the reactions to exclusion postulated by the need-threat model and the goal adjustment processes are part of rich research traditions that also have demonstrated such reactions in a variety of (mostly WEIRD) samples and application contexts (including real life, for ostracism see, e.g., Büttner et al., 2024; Nezlek et al., 2012; Rudert et al., 2020; for goal adjustment see introduction). The consistency of the current findings with this research suggests that the results are, in principle, generalizable. However, more research is needed, especially in non-WEIRD societies and in different real-life settings, to further explore the extent of generalizability and its limitations. In addition, individual differences in reactions, results regarding the different facets of GD, and results based on implicit measures, all of which have not been the subject of much research in ostracism research so far, require general replication efforts (including with similar samples). Finally, it should be mentioned that the study took place at a time when politically imposed social restrictions were in place in Germany as part of the coronavirus safety measures. The sample of the current study was therefore somewhat socially deprived, which may have increased the susceptibility to exclusion manipulation. However, numerous studies that have found strong effects of the exclusion manipulation even in non-deprived samples speak in favor of generalizability.

## Data Availability

The datasets presented in this study can be found in online repositories. The names of the repository/repositories and accession number(s) can be found at: https://doi.org/10.17605/OSF.IO/9JY6Q.

## Footnotes

^1^Additionally, we tested whether the Navon task, which was administered in the regulation phase (originally as a filler task), could be used as a sub-personal measure of attentional focus at that time. However, empirical results hinted at problems with the measure, so the details of this measure and the associated hypothesis tests (H12, H14) are not described in this paper for reasons of space. Instead, these analyses and further information can be found in the supplementary material on osf.

^2^ https://osf.io/w7svu/?view_only=56e06fe98b9040c1a571f1df3c88a982

^3^For a full list of all hypotheses in detail, see preregistration on osf.

^4^All Supplementary Material can be found at: https://osf.io/pb7cr/?view_only=24b68b84ddef4c879ea9d5e7136b34f0

^5^For a description of these measures see materials uploaded on the osf, dispositional goal disengagement and reengagement were measured by established scales (Wrosch, Scheier, Miller, et al., 2003).

^6^As in Rühs et al. (2022), we report uncorrected *p*-values in the Results section to allow for the use of different adjustment methods for multiple testing. We performed robustness-checks on our results using the conservative Bonferroni method to keep the family-wise type 1 error probability for each of the numbered hypotheses at 0.05. The number of tests considered per hypothesis varied from 1 to 12, so that the adjusted significance levels ranged from 0.05 to 0.004 (for details, see Supplementary Table 3 on osf). Even with this conservative approach, there were no changes in the decisions about the hypotheses.
